# Nurses’ knowledge to pressure ulcer prevention in public hospitals in Wollega: a cross-sectional study design

**DOI:** 10.1186/s12912-019-0346-y

**Published:** 2019-05-20

**Authors:** Werku Etafa Ebi, Getahun Fetensa Hirko, Diriba Ayala Mijena

**Affiliations:** 1 Department of Nursing, Institute of Health Science, Wollega University, P.O. Box 395, Nekemte, Ethiopia; 2 Department of Midwifery, Institute of Health Science, Wollega University, P.O. Box 395, Nekemte, Ethiopia

**Keywords:** Pressure ulcer, Prevention, Nurses, Knowledge

## Abstract

**Background:**

Pressure ulcer is a preventable medical complication of immobility. It has psychological, economic and social impact on individual and family. Its cost of treatment is more than twice of cost of prevention. It is primarily the nurses’ responsibility to prevent pressure ulcer. The aim of this study was to assess the nurses’ knowledge to pressure ulcer prevention in public hospitals in Wollega.

**Methods:**

A descriptive multicenter cross-sectional study design using quantitative method was employed to collect data from 212 randomly selected nurses. Data was collected using structured two validated self-administered instruments of pressure ulcer knowledge test evaluate nurses’ knowledge. Mean scores were compared using the Mann-Whitney U and Kruskal-Wallis tests. Means, standard deviation, and frequencies were used to describe nurses’ knowledge levels and barriers to pressure ulcer prevention.

**Results:**

Analysis of the study displayed 91.5% had inadequate knowledge to pressure ulcer prevention. The mean of nurses’ knowledge in all theme and per item were 11.31 (SD = 5.97) and 0.43 (SD = 0.22).respectively. The study participants had the highest mean item score (2.65 ± 0.87) in nutrition theme, whereas, scored lowest on etiology and development (0.27 ± 0.18) and preventive measures to reduce duration of pressure (0.29 ± 0.18), The study also identified significant nurses read articles (0.000) and received training (*p* = 0.003). Shortage of pressure relieving devices, lack of staff and lack of training were the most commonly cited perceived barriers to practice pressure ulcer prevention.

**Conclusions:**

This study highlights areas where measures can be made to facilitate pressure ulcer prevention in public hospitals in Wollega zones, such as increase regular adequate further training of nurses regarding pressure ulcer/its prevention points.

**Electronic supplementary material:**

The online version of this article (10.1186/s12912-019-0346-y) contains supplementary material, which is available to authorized users.

## Background

Pressure ulcers (PUs) prevention remains a significant challenge for nurses [1, 2], and its incidence is considered an indicator of poor quality of care [3–5]. Patients and families know that pressure ulcers are painful and slow to heal [5]. Some risk factors for the development of pressure ulcers/injuries include advanced age, immobility, incontinence, inadequate nutrition and hydration, neuro-sensory deficiency, device-related skin pressure, multiple comorbidities and circulatory abnormalities [5–7]. Ninety-five percent (95%) of pressure ulcers are avoidable [8, 9].

The incidence of pressure ulcers in adults varies from 0 to 12% in acute care settings, 24.3 to 53.4% in critical care settings and 1.9 to 59% in elderly care settings [6]. The prevalence of pressure ulcer has decreased over time in the USA (2004–2011 [10], 2006–2009 [11]). Two different cross-sectional studies conducted at Felegehiwot and Dessie referral hospital, in Ethiopia reported 16.8 and 14.9% overall prevalence rate of PU, respectively [12, 13]. Moreover, these studies identified risk factors PU such as prolonged hospitalization, slight limit of sensory perception, lack of regular positioning and activity, friction/shear [12, 13].

The cost for treating pressure ulcer increased proportionally to the increase of the area and the development of PU category [14]. PU treatment cost per patient per day varied between 2.65 € to 87.57€ across all settings and ranged from 1.71€ to 470.49€ across different settings [15].

When caregivers practice the best care every time, patients can avoid needless suffering [5]. Pressure area care is an essential component of nursing practice, with all patients potentially at risk of developing a pressure ulcer [16]. It is nurses’ primary responsibility for maintaining skin integrity [17, 18] and prevention of its complications [19]. Recognizing patients at risk of developing PU in early time is an essential part of the prevention care pathway [20]. The time nurses and healthcare assistant spent to patient care accounts for 90% of the overall costs for treating PUs, and 96% of the price in category I and II pressure ulcers [21].

Several studies have been undertaken to evaluate nurses’ knowledge to pressure ulcer prevention using different instruments, cutoff point and professional nurses (assistant, registered and students). A cross-sectional multicenter study [22] among nurses in Belgian hospitals reported that only 23.5% (130/553) of the nurses had scored ≥60% mean knowledge of pressure ulcer prevention. Demarr’e et al. [23] also displayed a low mean score (28.9%) of knowledge for registered nurses and nursing assistants (*n* = 145) in nursing home settings. In contrast, a survey in a Swedish healthcare setting among nursing staff showed that all respondents displayed good knowledge on prevention and treatment of pressure ulcers. Gunningberg et al. [24] studied prevention of PUs in a hospital wards and found more than half of the participants had a knowledge deficit (< 60% mean score).

Simonetti et al. [25] reported nursing students (*n* = 742) PU knowledge score below the mean (51.1%, 13.3/26) about PU prevention. Similarly, Qaddumi & Khawaldeh [26] found that the majority (73%) of Jordanian nurses had scored lower than the mean knowledge about pressure ulcer prevention. Meanwhile, nurses had scored the lowest in themes related to PU etiology, preventive measures to reduce amount of pressure/shear, and risk assessment.

Tirgari et al. [27] displayed Iranian Intensive Care Unit (ICU) nurses had score lower knowledge than the average, meanwhile, it showed the highest mean score and the lowest mean scores in theme etiology and development, classification and observation. Gul A et al. [28] found that among 308 nurses in an acute care Turkish hospital using modified and translated version of the Pieper PUKT most participants (58.4%) answered at least 60% of the questions correctly and scores were highest for the prevention/risk assessment and lowest for the PU staging domain. Using a multicenter cross-sectional study design Usher et al. [29] reported the overall mean knowledge score 51.1% which less than cutoff point (60%) among Australian nursing students. Similarly, it identified the lowest nursing students’ knowledge score on the themes preventive measures to reduce the amount of pressure/shear (44.1%) and the duration of pressure/shear (48.5%).

However, Panagiotopoulou and Kerr [30], found good level of knowledge among Greek nurses in relation to risk factors and areas at risk for pressure ulcer, with the average level of agreement with expert opinion being 70.5%. Similarly, Tweed and Tweed [31] evaluated critical care nurses’ knowledge level of pressure ulcer care using a testing tool developed specifically for that study and reported adequate knowledge to pressure ulcer prevention nursing staffs. A cross-sectional survey conducted among 248 nurses in Gondar University hospital using instrument developed by authors reported that early more than half (54.4%) of the nurses had good knowledge of PU prevention of [32].

Panagiotopoulou and Kerr [30], found that lack of staff/manpower (94. 9%), lack of equipment (78. 8%) and overcrowding in the ward (79.1%) as the most frequently identified nurses’ barriers to practice PU prevention. Similarly, Qaddumi & Khawaldeh [26] also measured lack of time (34.1%), shortage of staff (24.4%,), the patient’s condition (17.8%), and lack of resources or equipment (19.3%) as the major barriers nurses face to prevent pressure ulcer. Moore and Price [33] identified lack of staff and time meanwhile Kallman and Suserud [34] reported lack of time, equipment, resources, and patient condition are the most frequently cited barriers. The study at Mulago, Ugandan teaching hospital also found heavy workload related to shortage of staff (94.6%) and shortage of pressure relieving devices uncooperative patient (62.5%), poor access to pressure ulcer literature (37.5%) and inadequate coverage about pressure ulcers during training (23.2%) [35]. Samuriwo, & Dowding [36] indicated that nurses rely on their own knowledge and experience rather than research evidence to decide what skin care to deliver.

In Ethiopia, it not nurses’ culture to assess patients who are at risk or had developed PU before admitted to wards though PU is an emerging problem in developing counties in line with increasing population aging and the burden of chronic non-communicable disease. It is also obvious that there are limitations of resources used to enhance nurses’ knowledge and skill with updated evidence based works that could improve quality of nursing care in Ethiopia. For instance poor access to internet, absence of libraries to acquire reading materials (articles or updates about PU), limited in service training about PU or its prevention. Currently, there is no evidence on nurses’ knowledge regarding PU prevention in public hospitals in Wollega zones, West Ethiopia. Therefore, this cross sectional study was undertaken to assess nurses’ knowledge and perceived barriers to practice PU prevention.

## Objectives

The objective of this study was to evaluate nurses’ knowledge to PU prevention and to determine nurses’ perceived barriers to PU prevention in public hospitals in Wollega, Oromiya, Ethiopia.

## Methods

### Study design and setting

Institutional based cross-sectional multi-center study using quantitative method was conducted from August 13–22, 2018. Thera are 10 public hospitals functional in Wollega zones. The study setting includes five public hospitals including one teaching hospital (Wollega University Referral Hospital), five Public Referral Hospitals: Nekemte, Gimbi, Nedjo, and Shambu Referral hospitals. Among ten hospitals, the investigators purposively selected five hospitals where large number of patients visit, referred and admitted. Wollega’s main town (Nekemte) is 330 km to the west from the capital city of the country, Addis Ababa, Ethiopia. .

Swedish missionaries introduced the modern nursing to Ethiopia around 1866. Then, Russia and French were delivering the nursing service in limited areas of Ethiopia. After the Second World War (1949), the Ethiopian Red Cross Society established the first nursing school in at Haile Selassie I hospital. Swedish Missionaries at the Princess Tsehai Memorial Hospital opened the second nursing school. These two nursing schools were only admitting females to train in nursing profession. Males were admitted to nursing programs in 1954 in Ethiopia in Nekemte nursing school found in the current study area, Wollega zones. Currently, in Ethiopia nursing profession could be educated after completed grade ten (enjoy college to be enrolled in nursing assistants) or twelve (enjoy Universities and enrolled in actual diploma in nursing after 4 years completion of study in nursing profession) [37].

### Sample size and sampling procedure

The sample size was determined by using a single population proportion formula with the assumption of 54.4% Proportion [Gondar], 95% confidence level and 5% margin of error. Since the source of the population was less than 10,000 (*n* = 420), a correction formula was used. Using 10% nonresponse the final sample size obtained was 220. Then, the number of participants in each selected hospital to take a similar proportion of participants were determined using the proportionate population sampling.

### Study instrument

The questionnaire was administered in English language since it is a medium of instruction in nursing education in Ethiopia. A questionnaire used for data collection contained three parts (Additional file 1). Part one of the data collection was developed and included demographic characteristics such as gender, age, years of clinical experience in the nursing profession, level of current higher education, sources of PU knowledge, read articles about PU and training exposure to PU prevention.

Part two of data collection tool was Pressure Ulcer Knowledge Test Tool (PUKT), in an English version, to assess participant knowledge about pressure injuries that has acceptable reliability and validity, developed and validated by Beeckman et al. [38]. This instrument was validated for difficulty, discriminating index, and quality of the response alternatives. The internal consistency reliability (Cronbach’s α) was 0.77, and the 1-week test-retest interclass correlation coefficient (stability) was 0.88. Content validity index was 0.78 to 1.00. The item difficulty index of the questions ranged from 0.27 to 0.87, whereas values for item discrimination ranged from 0.29 to 0.65 [39].

The PUKT includes 26 multiple-choice questions in 6 categories: etiology and development (6), classification and observation (5), risk assessment (2), nutrition (1), preventive measures to reduce the amount of pressure (7), and preventive measures to reduce the duration of pressure (5) items. Each question has four answer options, and the fourth option is ‘I do not know the answer’ and scored zero points, which is included to prevent respondents from guessing the answer. Nurses who answered the item correctly scored one point, while who cannot answer correctly scored zero. This result in a final score between 0 and 26. Zero (0) and 26 scores represent nurses who incorrectly and correctly answered all nurses’ PU knowledge testing items from the total 26 items, respectively. The permission to use questionnaire communicated and obtained through the corresponding author electronic mail address from the corresponding author Beeckman [39]. Four nursing educators holding assistant professor and experienced researchers ensured the cultural and linguistic validity of instrument before the actual study conducted and determined the time required to complete filling the questionnaire after implemented comments.

The third part of the data collection tool was a list of barriers to the implementation of PU prevention. These instruments were adapted from the literature [26, 33, 35]. Some of items in the tool were modified as they are not applicable in Wollega nurses. Two types of options (‘Yes’ or ‘No’) were provided for nurses to select barriers that hinder nurses from exercising PU prevention points. It was used to identify nurses’ perceived barriers to practice PU prevention.

### Data collection

Firstly, we made contact with each hospital medical director and matron to grant a permission with a copy of approved ethical clearance letter obtained from Wollega University Department of Nursing Ethical Review Committee to undertake the study. All medical directors and matrons readily accepted our request. Secondly, the head nurses asked for their cooperation to give the permanent nurses staff list in their unit. Nurses who had no an experience of direct patient care, were on vacation, and employed and had clinical nursing experience less than 1 year were exclude from the study. Nurses from all units in each hospital who fulfill the inclusion criteria were included in the study.

In each hospital, matron were responsible for supervising the staff nurses participated in the study to ensure no resources/any references materials were needed. Two bachelors of Science degree nurses were responsible for participant recruitment and distribution of the questionnaire. Staff nurses were randomly selected from their list given using lottery method until the required number of nurses obtained. Data facilitators informed staff nurses about the study verbally, and distributed the participant information sheet and consent form to those who voluntarily agreed to participate.

The self-administered questionnaire was distributed to each nurse during working hours at each hospitals. Voluntary participant staff nurses were informed not use any resources or ask other staffs for answers while completing the questionnaire. Staff nurses who were not volunteer were permitted not to participate. Staff nurses were allowed to leave complete the questionnaire. The time estimated to complete the questionnaire was a minimum of 30 min.

### Data analysis

The data cleaning was done, entered into the computer using EPI data version 3.1 statistical packages, and checked for the consistency of data entry. The Statistical Package for Social Sciences (SPSS) version 20.0 (IBM Corporation, Armonk, NY) used for data analysis. Categorical variables computed as frequencies and percentages. Continuous variables compiled as mean and standard deviation (SD). The Mann- Whitney U and Kruskal-Wallis H tests were used to compare the mean score of independent groups. The statistical significance was set at *p*-value < 0.05.

## Results

### Sociodemographic characteristics of nurses

The total number of eligible nurses was 220; of these, 212 were volunteered to participate in the study, a response rate of 96.3%. Most of them were males (131, 61.8%). The mean age among the study participants was 28.2 ± 5.2 (range 21–54) years. Majority of study participants (148, 69.8%) were a diploma holder in nursing, 71.2% had 5–10 years of clinical experience in the nursing profession. One hundred sixty (160, 75.5%) of the participants attended education on PU; almost half (49.5%) of them got PU education at University/ college education. One hundred fifty-six (156, 73.6%) did not read articles about pressure ulcer, while, 138 (65.1%) of the participants had no exposure to PU training as illustrated in (Table 1).

**Table 1:** Demographic characteristics of the nurses (*N* = 212)

Variables	Frequency	Percentage
Gender		
Male	131	61.8
Female	81	38.2
Age (years)		
20–25	34	16
26–30	45	21.2
31–35	110	51.9
36–40	14	6.6
> 40	9	4.2
Current education level		
Diploma	148	69.8
Bachelor of science	64	30.2
Clinical experience in nursing (years)		
< 2	5	2.4
2–4	39	18.4
5–10	151	71.2
11–15	12	5.7
> =16	5	2.4
Source of education about PU		
University/College	105	49.5
Workplace	51	24.1
Conference/workshop	2	9
Articles	3	1.4
Never	51	24.1
Read researches /articles about PU		
Yes	56	26.4
No	156	73.6
Last attend training about PU		
Yes	92	34.9
No	138	65.1

### Nurses’ knowledge to prevent pressure ulcer

Analysis of knowledge items showed that the mean score of nurses’ knowledge about pressure ulcer prevention was 0.43 ± 0.22. Among the six categories of PU knowledge assessment, the nutrition category had the highest mean item score (2.65 ± 0.87), and etiology and development (0.27 ± 0.18) and preventive measures to reduce the duration of pressure (0.29 ± 0.18), had the lowest mean item score (Table 2). Similarly, Table 3 shows the percentage of nurses’ response to each question of the PUKT. The percentage of correct answers ranged from (133, 62.7%) to 31, 14.2%). The highest correct answers belonged to theme nutrition, item number 6, and multiple choice “c” ‘which said that optimizing nutrition can improve the patients’ general physical condition that may contribute to a reduction of the risk of pressure ulcers (62.7% answered correctly). The lowest scores (14.2%) of correct answers found under classification and observation theme, item number 7 which stated “A pressure ulcer extending down to the fascia is a grade 3 pressure ulcer.” More than 14 % (31, 14.6%) answered correctly item number 1, “lack of oxygen causes pressure ulcers” (Table 3).

**Table 2:** Mean and Standard Deviation (SD) of nurses’ knowledge score by categories

Knowledge Categories	Mean ± SD	Mean per item ± SD
Etiology and development (Items number1–6)	1.67 ± 1.10	0.27 ± 0.18
Classification and observations (Items number7–11)	2.12 ± 1.14	0.42 ± 0.22
Risk assessment (Items number 12–13)	0.90 ± 0.62	0.45 ± 0.31
Nutrition (Item number 14)	2.65 ± 0.87	2.65 ± 0.87
Preventive measures to reduce amount of pressure (Items number 15–21)	2.48 ± 1.32	0.35 ± 0.18
Preventive measures to reduce duration of pressure (Items number 22–26)	1.49 ± 0.92	0.29 ± 0.18
Total score	11.31 ± 5.97	0.43 ± 0.22

**Table 3:** Nurses’ knowledge to pressures ulcer prevention (*N* = 212)

Categories (Mean ± SD)	Items	Frequency of answers (%)
Etiology and development 1.67 ± 1.10	1. Which statement is correct?	
	a. Malnutrition causes pressure ulcers.	9 (4.2)
	b. A lack of oxygen causes pressure ulcers. ^a^	31 (14.6)
	c. Moisture causes pressure ulcers.	159 (75)
	d. I don’t’ know	13 (6.2)
	2. Extremely thin patients are more at risk of developing a pressure ulcer than obese patients.	
	a. The contact area involved is small and thus the amount of pressure is higher. ^a^	72 (34.0)
	b. The pressure is less extensive because the body weight of those patients is lower than the body weight of obese patients.	26 (12.2)
	c. The risk of developing a vascular disorder is higher for obese patients. This increases the risk of developing a pressure ulcer.	67 (31.6)
	d. I don’t’ know	47 (22.2)
	3. What happens when a patient, sitting in bed in a semi upright position (60-), slides down?	
	a. Pressure increases when the skin sticks to the surface.	41 (19.3)
	b. Friction increases when the skin sticks to the surface.	99 (46.7)
	c. Shearing increases when the skin sticks to the surface. ^a^	62 (29.3)
	d. I don’t’ know	10 (4.7)
	4. Which statement is correct?	
	a. Soap can dehydrate skin and thus the risk of pressure ulcers is increased.	5 (2.4
	b. Moisture from urine, feces, or wound drainage causes pressure ulcers.	160 (75.5)
	c. Shear is the force that occurs when the body slides and the skin sticks to the surface. ^a^	33 (15.5)
	d. I don’t’ know	14 (6.6)
	5. Which statement is correct?	
	a. Recent weight loss that has brought a patient below his/her ideal increases the risk of pressure ulcers. ^a^	73 (34.4)
	b. Very obese patients using medication that decreases the peripheral blood circulation are not at risk of developing pressure ulcers.	54 (25.5)
	c. Poor nutrition and age have no impact on tissue tolerance when the patient has a normal weight.	40 (18.9)
	d. I don’t’ know	45 (21.2)
	6. There is NO relationship between pressure ulcer risk and	
	a. Age.	58 (27.4)
	b. Dehydration.	48 (22.6)
	c. Hypertension. ^a^	83 (39.2)
	d. I don’t’ know	23 (10.8)
Classification and Observation 2.12 ± 1.14	7. Which statement is correct?	
	a. A pressure ulcer extending down to the fascia is a grade 3 pressure ulcer. ^a^	30 (14.2)
	b. A pressure ulcer extending through the underlying fascia is a grade 3 pressure ulcer.	50 (23.6)
	c. A grade 3 pressure ulcer is always preceded by a grade 2 pressure ulcer.	84 (39.6)
	d. I don’t’ know	48 (22.6)
	8. Which statement is correct?	
	a. A blister on a patient’s heel is always a pressure ulcer of grade 2.	9 (4.2)
	b. All grades (1, 2, 3, and 4) of pressure ulcers involve loss of skin layers.	28 (13.2)
	c. When necrosis occurs, it is a grade 3 or a grade 4 pressure ulcer. ^a^	137 (64.6
	d. I don’t’ know	38 (17.9)
	9. Which statement is correct?	
	a. Friction or shear may occur when moving a patient in bed. ^a^	81 (38.2)
	b. A superficial lesion preceded by non-blanchable erythema is probably a friction lesion.	56 (26.4)
	c. A kissing ulcer (coping lesion) is caused by pressure and shear.	49 (23.1)
	d. I don’t’ know	26 (12.3)
	10. In sitting position, pressure ulcers are most likely to develop on the:	
	a. Pelvic area, elbow, and heel. ^a^	146 (68.9)
	b. Knee, ankle, and hip.	23 (10.8)
	c. Hip, shoulder, and heel.	36 (17.3)
	d. I don’t’ know	7 (3.0)
	11. Which statement is correct?	
	a. All patients at risk of pressure ulcers should have a systematic skin inspection once a week.	16 (7.6)
	b. The skin of patients seated in a chair, who cannot move themselves, should be inspected every 2 to 3 h.	123 (58.0)
	c. The heels of patients who lie on a pressure-redistributing surface should be observed minimum a day. ^a^	56 (26.4)
	d. I don’t’ know	17 (8.0)
Risk assessment 0.90 ± 0.62	12. Which statement is correct?	
	a. Risk assessment tools identify all high-risk patients in need of prevention.	51 (24.1)
	b. The use of risk assessment scales reduces the cost of prevention.	32 (15.1)
	c. A risk assessment scale may not accurately predict the risk of developing a pressure ulcer and should be combined with clinical judgment. ^a^	92 (43.3)
	d. I don’t’ know	37 (17.5)
	13. Which statement is correct?	
	a. The risk of pressure ulcer development should be assessed daily in all nursing home patients.	17 (8.0)
	b. Absorbing pads should be placed under the patient to minimize the risk of pressure ulcer development.	77 (36.3)
	c. c. A patient with a history of pressure ulcers runs a higher risk of developing new pressure ulcers. ^a^	97 (45.8)
	d. I don’t know	21 (9.9)
Nutrition 2.65 ± 0.87	14. Which statement is correct?	
	a. Malnutrition causes pressure ulcers.	35 (16.5)
	b. The use of nutritional supplements can replace expensive preventive measures.	24 (11.4)
	c. Optimizing nutrition can improve the patients’ general physical condition that may contribute to a reduction of the risk of pressure ulcers. ^a^	133 (62.7)
	d. I don’t’ know	20 (9.4)
Preventive measures to reduce the amount of pressure 2.48 ± 1.32	15. The sitting position with the lowest contact pressure between the body and the seat is	
	a. An upright sitting position, with both feet resting on a footrest.	36 (17.0)
	b. An upright sitting position, with both feet resting on the floor.	77 (36.3)
	c. A backward sitting position, with both legs resting on a footrest. ^a^	67 (31.6)
	d. I don’t’ know	32 (15.1)
	16. Which repositioning scheme reduces pressure ulcer risk the most?	
	a. Supine position---side 90 lateral position---supine position---90 lateral position---supine position	62 (29.2)
	b. Supine position---side 30 lateral position---side 30 lateral position---supine position. ^a^	70 (33.0)
	c. Supine position---side 30 lateral position---sitting position---30 lateral position---supine position	49 (23.2)
	d. I don’t’ know	31 (14.6)
	17. Which statement is correct?	
	a. Patients who are able to change position while sitting should be taught to shift their weight minimum every 60 min while sitting in a chair.	29 (42.1)
	b. In a side-lying position, the patient should be at a 90 degree- angle with the bed.	125 (19.8)
	c. Shearing forces affect a patient’s sacrum maximally when the head of the bed is positioned at 30 degrees. ^a^	31 (20.8)
	d. I don’t’ know	27 (17.4)
	18. If a patient is sliding down in a chair, the magnitude of pressure at the seat can be reduced the most by	
	a. A thick air cushion. ^a^	92 (43.4)
	b. A donut-shaped foam cushion.	97 (45.8)
	c. A gel cushion.	14 (6.6)
	d. I don’t’ know	9 (4.2)
	19. For a patient at risk of developing a pressure ulcer, a viscoelastic foam mattress	
	a. Reduces the pressure sufficiently and does not need to be combined with repositioning.	29 (13.7)
	b. Has to be combined with repositioning every 2 h.^a^	125 (59.0)
	c. Has to be combined with repositioning every 4 h.	31 (14.6)
	d. I don’t’ know	27 (12.7)
	20. A disadvantage of a water mattress is	
	a. Shear at the buttocks increases.	49 (23.1)
	b. Pressure at the heels increases.	44 (20.8)
	c. Spontaneous small body movements are reduced. ^a^	69 (32.5)
	d. I don’t’ know	50 (23.6)
	21. When a patient is lying on a pressure-reducing foam mattress,	
	a. Elevation of the heels is not necessary.	29 (13.7)
	b. Elevation of the heels is important. ^a^	62 (29.3)
	c. He/she should be checked for “bottoming out” at least twice a day.	94 (44.3)
	d. I don’t’ know	27 (12.7)
Preventive measures to reduce the duration of pressure 1.49 ± 0.92	22. Repositioning is an accurate preventive method because	
	a. The magnitude of pressure and shear will be reduced.	50 (23.6)
	b. The amount and the duration of pressure and shear will be reduced.	99 (46.7)
	c. The duration of pressure and shear will be reduced. ^a^	36 (17.0)
	d. I don’t’ know	27 (12.7)
	23. Fewer patients will develop a pressure ulcer if	
	a. Food supplements are provided.	22 (10.4)
	b. The areas at risk are massaged.	121 (57.1)
	c. Patients are mobilized. ^a^	34 (16.0)
	d. I don’t’ know	35 (16.5)
	24. Which statement is correct?	
	a. Patients at risk lying on a non-pressure-reducing foam mattress should be repositioned every 2 h.^a^	69 (32.5)
	b. Patients at risk lying on an alternating air mattress should be repositioned every 4 h.	58 (27.4)
	c. Patients at risk lying on viscoelastic mattress should be repositioned every 2 h.	56 (26.4)
	d. I don’t’ know	29 (13.7)
	25. When a patient is lying on an alternating air mattress, the prevention of heel pressure ulcers includes	
	a. No specific preventive measures.	11 (5.2)
	b. A pressure-reducing cushion under the heels.	83 (39.2)
	c. A cushion under the lower legs elevating the heels. ^a^	94 (44.3)
	d. I don’t’ know	24 (11.3)
	26. If a bedridden patient cannot be repositioned, the most appropriate pressure ulcer prevention is	
	a. A pressure-redistributing foam mattress.	55 (25.9)
	b. An alternating-pressure air mattress. ^a^	83 (39.2)
	c. Local treatment of the risk areas with zinc oxide paste.	49 (23.1)
	d. I don’t’ know	25 (11.8)

^a^ Indicates correct answers for each question

Nurses’ knowledge score to PU were higher among those who read articles about PUs (*P* = .000) and attended training in the last (*P* = .003). Similarly, there is a statistically significant difference in knowledge score among gender (*p* = 0.000). The study identified variables such as gender, age, level of education, clinical experience in the nursing profession and source of education had no significant difference in knowledge score (Table 4).

**Table 4:** Demographic variables association

Variables	Frequency	Percentage	p-value
Gender			0.00^a^
Male	131	61.8	
Female	81	38.2	
Age (years)			0.60
20–25	34	16	
26–30	45	21.2	
31–35	110	51.9	
36–40	14	6.6	
> 40	9	4.2	
Current education level			0.72
Diploma	148	69.8	
Bachelor of science	64	30.2	
Clinical experience in nursing (years)			0.20
< 2	5	2.4	
2–4	39	18.4	
5–10	151	71.2	
11–15	12	5.7	
> =16	5	2.4	
Source of education about PU			0.33
University/College	105	49.5	
Workplace	51	24.1	
Conference/workshop	2	9	
Articles	3	1.4	
Never	51	24.1	
Read research studies/articles about PU?			0.00^a^
Yes	56	26.4	
No	156	73.6	
Last attend training about PU			0.03^a^
Yes	92	34.9	
No	138	65.1	

^a^indicates significant difference between variables

*PU* Pressure ulcer

### Nurses’ perceived barriers to implement pressure ulcer prevention

A descriptive analysis identified the most common barriers of nurses to practice pressure ulcer prevention; Lack of staff/heavy workload (116, 54.7%), shortage of pressure relieving devices (117, 55.2%), lack of training (110, 51.9) and lack of multidisciplinary initiative (101, 47.6%) (Table 5).

**Table 5:** Nurses’ barriers to practice Pressure ulcer prevention (*N* = 212)

Variables	Frequency (%)
Lack of staff/heavy workload	116 (54.7)
Lack/ poor opportunities to update knowledge	89 (42)
Lack of universal guideline	90 (42.5)
Shortage of pressure relieving devices	117 (55.2)
Poor risk assessment tool skill	77 (36.3)
Seriously ill/uncooperative patient	93 (43.9)
Lack of training	110 (51.9)
Lack of job satisfaction	74 (34.9)
Lack of multidisciplinary initiative	101 (47.6)
Others	17 (8.0)

## Discussion

The present study used a multicenter cross-sectional design aims to investigate the knowledge of nurses about pressure ulcer prevention in Wollega public hospitals and to identify nurses’ barriers to practice pressure ulcer prevention. The result displayed that the knowledge of nurses about pressure ulcer prevention in Wollega hospitals was poor. It showed only 18 (8.5%) of nurses scored above the mean score (answered 13 out of 26). Our study reported relatively lower mean knowledge score (0.43), in agreement with Tirgari et al. [27], who conducted study among 89 Iranian intensive critical care nurses and reported the mean score of pressure injury knowledge 0.44 using the same instrument.

However, our scores are lower than the result reported from similar studies and the same instruments of measurement. For instance, Qaddumi & Khawaldeh [26] using the same cutoff point showed Jordanian nurses are more knowledgeable about PU prevention than nurses in working in public hospitals in Wollega. Similarly, a multicenter study conducted by Beeckman et al. [22] among 533 Belgian nurses found a knowledge score of 49.6% using 60% as cutoff point using the same instrument. Additionally, Simonetti et al. [25] among seven schools of Italian nursing students reported relatively lower knowledge scores (51%) using the same cutoff point with Beeckman et al. [22].

Moreover, our scores are also lower than those reported from similar studies using different instrument (Pressure Ulcer Knowledge Assessment Tool) of measurement. Gunningberg et al. [24] displayed that a knowledge score of 61.0% for staff nurses, 59.3% for registered nurses and 55.4% for assistant nurses in Sweden. Demarré et al. [23] study result among 145 registered and assistant nurses reported unsatisfactory level (28.9%) in nursing home settings.

In the present study, nurses’ gender (*p* = 0.000), nurses read articles (*p* = 0.001) and last attended training (*p* = 0.003) showed a significant difference to PU knowledge score. Qaddumi & Khawaldeh [26] in line with this study reported a significant difference between gender (male, 5.67% and female, 3.3%, *p* = 0.021). Tiragari et al. [27], Hulsenboom et al. [38], Li Z et al. [40], Kaddourah et al. [41] displayed nurses’ age is statistically significant to PU knowledge score in opposite to this study.

Our study also explained nurses’ knowledge score has no significant difference between education level (*p* = 0.72). However, some studies [32, 38, 42] report indicates a higher knowledge score among those completed higher education. Similarly, Simonetti et al. [25] nursing students’ year of education (*p* = < 0.001) and the number of department frequented during their clinical placement (*p* = 0.001) were significantly related to knowledge score.

Moreover, the current study also showed nurses’ sources of education about PU prevention and clinical nursing experience had no significant difference to PU knowledge score by nurses. Meanwhile, it determined nurses who read articles about PU and receive training about PU had higher knowledge score than those who did not read and attended training about PU. Meanwhile, Beeckman et al. [39] explained nurses who attended additional training displayed higher knowledge scores than nurses who did not attend any additional training (*p* = .002). Hulsenboom et al. [38] addressed nurses’ work experience, and educational level displayed higher knowledge score; Liz et al. [40] evaluated nurses with longer employment duration, previous training experience and who work in tertiary hospitals or critical care had higher knowledge score. A survey of Turkish nurses stated higher knowledge sore among nurses who read articles/books about PUs (*P* = .002), and who had attended at lecture/conference/course on PUs in the past [28]. Tiragari et al. [27] in opposition to the current study result reported a significant difference between previous exposures to PU education.

Failing to practice prevention is not restricted to inadequate knowledge. Study finding also suggested that the common barriers such as shortage of pressure relieving devices (117, 55.25%), lack of staff/heavy workload (116, 54.7%), lack of training (110, 51.9%) and lack of multidisciplinary initiative (101, 47.6%). Shortage of pressure relieving devices are the most frequently cited perceived barriers for nurses to practice PU prevention in this study. From our experience, our country, Ethiopia, is a developing country, and medical equipment supplied for health institutions are insufficient. Requesting and using appropriate equipment, using turning charts or upgrading mattress can all be put in place as preventive measures [6].

The second nurses’ perceived barrier to carrying out PU prevention identified in this study is lack of staff/heavy workload. The time nurses and healthcare assistant spent on patient care accounts for the highest cost of PU treatment [21]. Qaddumi and Khawaldeh [26], recommended nurses give priority to other illness rather than PU prevention care and complain PU care as an interdisciplinary problem when staff shortages with the stress happened. In our country, Ethiopia, due to low economy the number of nurses employed in hospitals are less than required number. This cause nurses not to spend plenty of at bedside. To the authors’ knowledge, there is no fixed rule for the patient to nurse ratio in the study area.

Lack of training also mentioned among the ordinary nurses’ perceived barriers. Regular training courses and review of PI prevention guidelines can be useful in updating the knowledge of nurses on pressure injury prevention [43]. Furthermore, Keast et al. [44] recommended educational programs for the prevention of PUs should be structured, organized, and comprehensive and should be updated on a regular basis to incorporate new evidence and technologies. In our country, Ethiopia, PU there is no programmed training and formulated guideline about PU prevention.

Saleh et al. [45] stated “PU education program as a powerful tool for nurses to improve understanding of PU, keep abreast of current knowledge on PU, and eliminate patient’s suffering”. Feng et al. [46] suggested that an education programme for PU prevention not only show an increase in staff knowledge also it leads to a significant decrease in incidents of PUs. Lack of multidisciplinary initiative is another nurses’ obstacle to put into practice PU prevention. PU prevention needs multidisciplinary efforts and teamwork to contribute to successful care [26]. PU prevention practice is not only the nurses’ responsibility though it is an integral part of intensive care nursing [47].

## Limitations

The study presents some limitations that need to be considered. Randomly selected participants may have been less motivated to complete the knowledge questionnaire, and the results might be too poor. Pretest was not conducted given the importance to use validate instruments. In addition, though nurses were informed not to exchange ideas and answers, diffuse information with each other and not to refer textbooks when completing the questionnaire, we have no guarantee if they complied with this instruction. However, we believed that the result of this study could be generalizable to all nurses working in the public hospitals in Wollega since the similar type of nursing education they receive and PU prevention points they practice.

## Conclusions

This study demonstrates that majority of the nurses have insufficient knowledge to practice pressure ulcer prevention. Nurses who read articles reading articles and attended training in the showed a significant difference to PU knowledge score. Shortage of pressure relieving devices, lack of staff/heavy workload and inadequate training were the most frequently cited nurses’ barriers to practice PU prevention. Providing opportunity to access resources (readable about and pressure reliving devices to) PU prevention, in-service training/regular training, incorporating and prioritizing in nursing curriculum, and formulating guidelines are some of the primary points to enhance nurses’ knowledge about pressure ulcer prevention. Further research on using observational studies is needed to determine the actual rather than the perceived practice to PU prevention.

## Additional file

Additional file 1: Data collection tool. (DOCX 21 kb)

## Abbreviations

ICU: Intensive Care Unit
PU: Pressure Ulcer
PUKT: Pressure Knowledge Test
SD: Standard Deviation
SPSS: Statistical Package for Social Sciences
USA: Unites States of America
