# Supplementary material for: Nurses’ knowledge to pressure ulcer prevention in public hospitals in Wollega: a cross-sectional study design
Source: BMC Nurs. 2019 May 20;18:20. doi: 10.1186/s12912-019-0346-y (PMC6528293; doi:10.1186/s12912-019-0346-y)
Supplement: Supplementary file 1 — Data collection tool. (DOCX 21 kb) [file 12912_2019_346_MOESM1_ESM.docx]

Questionnaire used to assess nurses’ knowledge to pressure ulcer prevention among nurses working in public hospitals in Wollega. This questionnaire has three parts. Time allocated to complete the questionnaire is 30 minutes.

| Part One: Demographic Information  Instruction: Encircle to the number that contain your correct answer or put your answer on the space provided (the most appropriate answer). | | | |
| --- | --- | --- | --- |
| S/No- | Questions | Response | Code |
| 1 | What is your gender? | 1. Male 2. Female | S1 |
| 2 | How old are you? | ________________(in years) | S2 |
| 3 | What is your current level of education? | 1. Diploma in nursing 2. Degree in nursing 3. Masters in nursing | S3 |
| 4 | How long is your clinical experience in nursing profession? | __________________(year/s) | S4 |
| 5 | Which was your source of education about pressure ulcer? | 1. University/College 2. Workplace 3. Conference/workshop 4. Reading articles 5. Never | S5 |
| 6 | Did you read articles about pressure ulcer? | 1. Yes 2. No | S6 |
| 7 | Have you ever attended training about pressure ulcer? | 1. Yes 2. No | S7 |

Part 2: Tools used to assess nurses’ knowledge to pressure ulcer prevention

Instruction: Choose the best answer among the given alternatives for each questions. Encircle to your correct response.

Theme 1: Etiology and development

1. Which statement is correct?
2. Malnutrition causes pressure ulcer
3. A lack of oxygen causes pressure ulcers.
4. Moisture causes pressure ulcers
5. I don’t’ know
6. Extremely thin patients are more at risk of developing a pressure ulcer than obese patients.
7. The contact area is small and thus the amount of pressure is higher.
8. The pressure is less extensive because the body weight of those patients is lower than the body weight of obese patients.
9. The risk of developing a vascular disorder is higher for obese patients which increases the risk of developing a pressure ulcer
10. I don’t’ know
11. What happens when a patient, sitting in bed in a semi-upright position (60°), slides down?
12. Pressure increases when the skin sticks to the surface.
13. Friction increases when the skin sticks to the surface.
14. Shearing increases when the skin sticks to the surface.
15. I don’t’ know
16. Which statement is correct?
17. Soap can dehydrate skin and thus the risk of pressure ulcers is increased.
18. Moisture from urine, feces, wound drainage causes pressure ulcers.
19. Shear is the force which occurs when the body slides and the skin sticks to the surface.
20. I don’t’ know
21. Which statement is correct?
22. Recent weight loss which has brought a patient below his or her ideal weight increases the risk of pressure ulcers.
23. Very obese patients using medication that decreases peripheral blood circulation are not at risk of developing pressure ulcers.
24. Poor nutrition and age have no impact on tissue tolerance when the patients have a normal weight.
25. I don’t’ know
26. There is NO relationship between pressure ulcers risk and:
27. Age
28. Dehydration
29. Hypertension
30. I don’t’ know

Theme 2: Classification and observation

1. Which statement is correct?
2. A pressure ulcer extending down to the fascia is a grade 3 pressure ulcer.
3. A pressure ulcer extending through the underlying fascia is a grade 3 pressure ulcer.
4. A grade 3 pressure ulcer is always preceded by a grade 2 pressure ulcer.
5. I don’t’ know
6. Which statement is correct?
7. A blister on a patient's heel is always a pressure ulcer of grade 2.
8. All grades (1, 2, 3 and 4) of pressure ulcers involve loss of skin layers.
9. When necrosis occurs, it is a grade 3 or grade 4 pressure ulcers.
10. I don’t’ know
11. Which statement is correct?
12. Friction or share may occur when moving a patient in bad.
13. A superficial lesion, preceded by non-blanch able erythema is probably a friction lesion.
14. A kissing ulcer (copy lesion) is caused by pressure and shear.
15. I don’t’ know
16. In a sitting position, pressure ulcers are more likely to develop on:
17. Pelvic area, elbow and heel.
18. Knee, ankle and hip.
19. Hip, shoulder and heel.
20. I don’t’ know
21. Which statement is correct?
22. All patients at risk of pressure ulcers should have a systematic skin inspection once a week.
23. The skin of patients seated in a chair, who cannot move themselves, should be inspected every 2 to 3 h.
24. The heels of patients who lie on a pressure redistributing should be observed minimum a day.
25. I don’t’ know

Theme 3: Risk assessment

1. Which statement is correct?
2. Risk assessment tools identify all high risk patients in need of prevention.
3. The use of need assessment scale reduces the cost of prevention.
4. A risk assessment scale may not accurately predict the risk of developing a pressure ulcer and should be combined with clinical judgment.
5. I don’t’ know
6. Which statement is correct?
7. The risk of pressure ulcer development should be assessed daily in all nursing home patients.
8. Absorbing pads should be placed under the patient to minimize the risk of pressure ulcer development.
9. A patient with a history of pressure ulcers runs a higher risk of developing new pressure ulcers.
10. I don’t’ know

Theme 4: Nutrition

1. Which statement is correct?
2. Malnutrition causes pressure ulcers.
3. The use of nutritional supplements can replace expensive preventive measures.
4. Optimizing nutrition can improve the patients' general physical condition
5. I don’t’ know

Theme 5: Preventive measures to reduce the amount of pressure/shear

1. The sitting position with the lowest contact pressure between the body and the seat is
2. An upright sitting position, with both feet resting on a footrest.
3. An upright sitting position, with both feet resting on the floor.
4. A backwards sitting position, with both legs resting on a footrest.
5. I don’t’ know
6. Which repositioning scheme reduces pressure ulcer risk the most?
7. Supine position–side 90° lateral position–supine position–90° lateral position–supine position
8. Supine position–side 30° lateral position–side 30° lateral position–supine position
9. Supine position–side 30° lateral position–sitting position–30° lateral position–supine position
10. I don’t’ know
11. Which statement is correct?
12. Patientswho are able to change positionwhile sitting should be taught to shift their weight minimum every 60 min while sitting in a chair.
13. In a side lying position, the patient should be at a 90 degree angle with the bed.
14. Shearing forces affect a patient sacrum maximally when the head of the bed is positioned at 30°.
15. I don’t’ know
16. If a patient is sliding down in a chair, the magnitude of pressure at the seat can be reduced the most by:
17. A thick air cushion.
18. A donut shaped foam cushion.
19. A gel cushion.
20. I don’t’ know
21. For a patient at risk of developing a pressure ulcer, a visco-elastic foam mattress:
22. Reduces the pressure sufficiently and does not need to be combined with repositioning.
23. Has to be combined with repositioning every 2 h.
24. Has to be combined with repositioning every 4 h.
25. I don’t’ know
26. A disadvantage of a water mattress is:
27. Shear at the buttocks increases.
28. Pressure at the heels increases.
29. Spontaneous small body movement is reduced.
30. I don’t’ know
31. When a patient is lying on a pressure reducing foam mattress:
32. Elevation of the heels is not necessary.
33. Elevation of the heels is important.
34. He or she should be checked for “bottoming out” at least twice a day.
35. I don’t’ know

Theme 6: Preventive measures to reduce the duration of pressure/shear

1. Repositioning is an accurate preventive method because…
2. The magnitude of pressure and shear will be reduced.
3. The amount and the duration of pressure and shear will be reduced.
4. The duration of pressure and shear will be reduced
5. Fewer patients will develop a pressure ulcer if:
6. Food supplements are provided.
7. The areas at risk are massaged.
8. Patients are mobilized.
9. I don’t’ know
10. Which statement is correct?
11. Patients at risk lying on a non-pressure reducing foam mattress should be repositioned every 2 h.
12. Patients at risk lying on an alternating air mattress should be repositioned every 4 h.
13. Patients at risk lying on a visco-elastic foam mattress should be repositioned every 2 h.
14. I don’t’ know
15. When a patient is lying on an alternating pressure air mattress, the prevention of heel pressure ulcers includes:
16. No specific preventive measures.
17. A pressure reducing cushion under the heels.
18. A cushion under the lower legs elevating the heels.
19. I don’t’ know
20. If a bedridden patient cannot be repositioned, the most appropriate pressure ulcer prevention is:
21. A pressure redistributing foam mattress.
22. An alternating pressure air mattress.
23. Local treatment of the risk areas with zinc oxide paste
24. I don’t’ know

Part 3: What are the challenges that hinder you from proper prevention of pressure ulcer? Please put the tick mark “√”in the corresponding column

| Perceived barriers | Yes | No |
| --- | --- | --- |
| Lack of staff/heavy workload |  |  |
| Lack/ poor opportunities to update knowledge |  |  |
| Lack of universal guideline |  |  |
| Shortage of pressure relieving devices |  |  |
| Poor risk assessment tool skill |  |  |
| Seriously ill/uncooperative patient |  |  |
| Lack of training |  |  |
| Lack of job satisfaction |  |  |
| Lack of multidisciplinary initiative |  |  |
| Others |  |  |
